# Effects of sensory manipulations on locomotor adaptation to split-belt treadmill walking in healthy younger and older adults

**DOI:** 10.1016/j.ibneur.2022.01.007

**Published:** 2022-02-01

**Authors:** Daniel Kuhman, Alyson Moll, William Reed, Noah Rosenblatt, Kristina Visscher, Harrison Walker, Christopher P. Hurt

**Affiliations:** a Rehabilitation Science, University of Alabama at Birmingham, Birmingham, AL, USA; b Physical Therapy, University of Alabama at Birmingham, Birmingham, AL, USA; c Dr. William M. Scholl College of Podiatric Medicine’s Center for Lower Extremity Ambulatory Research (CLEAR), Rosalind Franklin University of Medicine and Science, North Chicago, IL, USA; d Department of Neurobiology, University of Alabama at Birmingham School of Medicine, Birmingham, AL, USA; e Department of Neurology, University of Alabama at Birmingham School of Medicine, Birmingham, AL, USA

**Keywords:** Aging, Gait, Adaptation, Motor Control, Locomotion

## Abstract

Locomotor adaptation relies on processes of both the peripheral and central nervous systems that may be compromised with advanced age (e.g., proprioception, sensorimotor integration). Age-related changes to these processes may result in reduced rates of locomotor adaptation under normal conditions and should cause older adults to be disproportionately more affected by sensory manipulations during adaptation compared to younger adults. 17 younger and 10 older adults completed five separate 5-minute split-belt walking trials: three under normal sensory conditions, one with 30% bodyweight support (meant to reduce proprioceptive input), and one with goggles that constrained the visual field (meant to reduce visual input). We fit step length symmetry data from each participant in each trial with a single exponential function and used the time constant to quantify locomotor adaption rate. Group by trial ANOVAs were used to test the effects of age, condition, and their interaction on adaptation rates. Contrary to our hypothesis, we found no evidence that sensory manipulations disproportionately affected older compared to younger adults, at least in our relatively small sample. In fact, in both groups, adaptation rates remained unaffected across all trials, including both normal and sensory manipulated trials. Our results provide evidence that both younger and older adults were able to adequately reweight sources of sensory information based on environmental constraints, indicative of well-functioning neural processes of motor adaptation.

## 1 Introduction

Human locomotion often occurs in complex environments (e.g., uneven surfaces, obstacles, altered terrain, etc.). Successfully negotiating such environments requires the ability to adapt gait patterns to changing locomotor demands (i.e., locomotor adaptation). Locomotor adaptation relies on neural processing of information about ongoing movement. This processing includes aspects of both peripheral and central nervous systems, as individuals must accurately sense changes in the environment, integrate feedback across multiple sensory sources, and update gait patterns appropriately – known broadly as sensorimotor integration (for a review, see (Hinton, Conradsson, & Paquette, 2020)). An important feature of sensorimotor integration is sensory reweighting – the ability to “weight” sources of sensory information based on their relative importance/reliability during a motor task (Assländer and Peterka, 2014, Kabbaligere et al., 2017, Peterka, 2002). Maintenance of gait adaptability in the face of unreliable feedback from one or more sensory sources during a walking task can provide insight into one’s ability to engage sensory reweighting and thus the overall health of the sensorimotor integration system. Aging has been shown to reduce sensorimotor processing, affecting performance on novel complex motor tasks (Berard et al., 2009, Bugnariu and Fung, 2006). However, the degree to which age-related deficits in sensorimotor processing affect locomotor adaptation remain unclear.

Split-belt treadmill walking protocols are commonly used to assess locomotor adaptation. These protocols involve walking on a treadmill with two parallel, independently controlled belts, with one foot on each belt (Dietz et al., 1994, Reisman et al., 2005, Vasudevan et al., 2017). Initially, both belts move at the same speed (“tied-belt”), allowing individuals to rely on a feedforward control model in a predictable and somewhat normal movement environment. The belts then move at different speeds, with one belt faster than the other (“split-belt”), for a prolonged period. Immediately after the split-belt condition is introduced, sensory feedback no longer aligns with the feedforward model’s previous predictions, triggering a sensory-prediction error (Morton & Bastian, 2006). In response, individuals initially exhibit a large between-limb asymmetry in step length. Over several minutes of continuous split-belt walking, individuals gradually restore step length symmetry as they recalibrate their feedforward model of control for the split-belt environment (Bruijn et al., 2012, Morton and Bastian, 2006, Reisman et al., 2005, Reisman et al., 2007). The rate at which step length symmetry is restored following the introduction of the split-belt condition represents the rate at which the nervous system integrates sensory feedback and “updates” motor output (i.e., it provides a behavioral measure of sensorimotor integration). Split-belt treadmill walking, coupled with sensory manipulations, provides an opportunity to assess whether individuals can engage sensory reweighting to maintain adaptive behavior and may provide a non-invasive, behavioral assessment of sensorimotor integration.

Advanced age has been associated with dysfunctional sensorimotor integration, which has negative consequences for motor adaptation and limit the ability of older adults to safely and independently negotiate complex locomotor environments (i.e., reduced mobility) (Berard et al., 2009, Degardin et al., 2011). Although several previous studies have tested the effects of age on split-belt adaptation, to our knowledge none have combined split-belt walking with sensory manipulations to assess whether healthy older adults can engage sensory reweighting to maintain gait adaptability (Bruijn et al., 2012, Malone and Bastian, 2016, Vervoort et al., 2019). The primary purpose of this study was to assess the effects of sensory manipulations on locomotor adaptation in both young and old adults. Specifically, we tested the effects of bodyweight support (meant to reduce proprioceptive feedback (Jensen, Prokop, & Dietz, 1998)) and restricted vision (limited peripheral vision) on locomotor adaptation to split-belt treadmill walking. We hypothesized that sensory manipulations would have disproportionately larger effects on locomotor adaption in old compared to young adults, likely due to age-related dysfunction of neural processes involved in motor adaptation – particularly sensorimotor integration.

## 2 Materials and methods

### 2.1 Participants

17 healthy young adults (mean (SD): age = 24.4 (2.9) years) and 10 healthy old adults (age = 71.7 (4.5) years) were enrolled in this study. As part of the inclusion criteria, we enrolled young adults between the ages of 18–35 and old adults between the ages of 60–84. Exclusion criteria included self-reported inability to walk independently for at least 8 min, lower extremity injury in the previous six months, history of lower extremity joint surgery, and any neuromuscular or orthopedic disorder that could limit one’s ability to walk independently. No a priori power calculation was performed, and issues related to COVID-19 made it difficult to recruit and enroll participants, particularly for our older cohort, resulting in a relatively small sample of participants. This study was approved by the University of Alabama at Birmingham Institutional Review Board. All individuals provided written informed consent prior to performing the protocol.

### 2.2 Experimental protocol

**Fig. 1:**
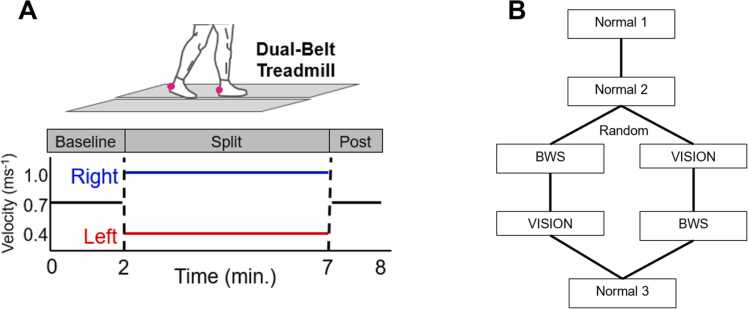
Schematic representations of the split-belt protocol (panel A) and the order of experimental conditions (B).

Reflective markers were secured to each heel of each participant to quantify step length. Participants then performed five split-belt treadmill walking trials with the following structure: 2 min with both belts tied at 0.7 ms^−1^ (“baseline”), 5 min with the left belt at 0.4 ms^−1^ and the right belt at 1.0 ms^−1^ (“split-belt”), and 1 min with both belts tied at 0.7 ms^−1^ (“post-adaption”). Thus, each trial lasted 8 min in total. This protocol was performed under normal (always trials 1, 2, and 5), visually restricted (VISION; randomized to trial 3 or 4), and bodyweight supported (BWS; randomized to trial 3 or 4) conditions (Fig. 1). We chose to standardize the order of normal trials to account for potential between-group differences in initial adaptation between younger and older adults. Having all participants complete the first two trials under normal sensory conditions ensured that both groups had been familiarized with the task so that data from sensory manipulated trials could be more confidently interpreted as the result of the manipulations rather than continued task learning from trial to trial. In “normal” trials, participants completed the protocol with full vision and no external BWS (however, individuals wore a harness secured to an overhead railing to ensure safety in the case of a fall). Multiple normal trials were included in the protocol to account for potential trial-to-trial carry-over effects prior to comparing data from normal and sensory manipulated conditions (Malone, Vasudevan, & Bastian, 2011). In the visually restricted condition, individuals wore a pair of goggles (Fork in the Road Vision Rehabilitation Services, LLC, WI, USA) that constricted their visual field to 20° (as if to simulate early-stage glaucoma or retinitis pigmentosa) and were explicitly instructed to avoid looking at their feet, which was confirmed visually by a member of the research team during testing (individuals did not receive external BWS but wore the same safety harness described above). In the BWS condition, we provided 30% BWS via an overhead harness system connected to a trolley located directly above the treadmill (individuals had full vision). We chose 30% BWS because this level of support has been shown by others to induce observable changes in split-belt adaptation (Jensen et al., 1998). The BWS system moves smoothly along a rail spanning the length of the treadmill, allowing individuals to move freely fore-aft. During all trials, individuals were specifically instructed to avoid touching the handrails located alongside the treadmill – this was confirmed visually by the research team during data collection; if at any point participants contacted the handrail, they were instructed to remove their hand immediately. Between trials, participants exited the treadmill and were encouraged to walk freely around the lab for at least 5 min (however participants were allowed to rest long if desired, in case of fatigue). After the between-trial “rest” period, all participants were asked to verbally confirm they felt that their gait had returned to normal. Thus, in total, all participants received at least 8 min of either tied-belt or overground walking between split-belt exposures (1 min tied-belt walking during post-adaptation, at least 5 min of overground walking, and 2 min of tied-belt walking during the baseline phase of the subsequent trial).

Kinematic data of reflective markers were collected using 9 infrared cameras (Vicon Motion Systems, Denver, CO, USA; 100 Hz) while individuals walked on the dual-belt treadmill (Motek Link, Amsterdam, Netherlands). Custom D-Flow (Motek Link) software was used to automate the split-belt treadmill walking protocol; Vicon Nexus software was used to collect and process kinematic data; laboratory software written in MATLAB (MathWorks Inc., Natick, MA, USA) and R were used for analysis.

### 2.3 Data analysis

Three-dimensional heel marker trajectories were filtered using a 4th-order lowpass Butterworth filter with a cutoff frequency of 6 Hz. Like previous split-belt treadmill walking studies, we used step length symmetry to explore locomotor adaptation. Step length (SL) was defined as the anteroposterior distance between the two heel markers at heel strike of each step. SL symmetry was defined as:

$${\mathrm{SL}}_{\mathrm{sym}\_\mathrm{raw}}=\frac{\left({\mathrm{SL}}_{\mathrm{fast}}-\;{\mathrm{SL}}_{\mathrm{slow}}\right)}{\left({\mathrm{SL}}_{\mathrm{fast}}+\;{\mathrm{SL}}_{\mathrm{slow}}\right)}$$

Where SL_fast_ and SL_slow_ refer to SL calculated when the leading leg is on the faster (right) and slower (left) moving belt, respectively. From this equation, an SL_sym_ value of zero represents perfect spatial symmetry between legs. However, to account for potential differences in baseline symmetries and step-to-step variability in symmetry between participants and/or across conditions within a participant, we transformed SL_sym_ values into z-scores:

$${\mathrm{SL}}_{\mathrm{sym}}\left(n\right)=\;\frac{{\mathrm{SL}}_{\mathrm{sym}\_\mathrm{raw}}\left(n\right)-\mathrm{mean}({\mathrm{SL}}_{\mathrm{sym}\_\mathrm{raw},\;\mathrm{baseline}})}{\mathrm{SD}({\mathrm{SL}}_{\mathrm{sym}\_\mathrm{raw},\;\mathrm{baseline}})}$$

Where n represents stride number; mean and standard deviation (SD) are calculated using SL_sym_ across the entire baseline phase. This z-score transformation was performed under the assumption that individuals adapt SL_sym_ back toward baseline norms rather than toward perfect spatial symmetry.

**Fig. 2:**
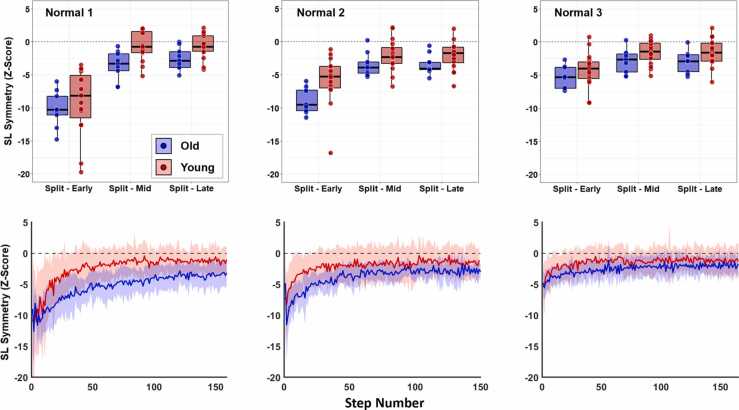
SL_sym_ bins (top row of panels) and step-by-step SL_sym_ data traces (bottom row of panels) for all three normal trials. In all panels, blue data represents old and red data represents young adults. In step-by-step data, solid lines represent group mean and shaded areas represent standard deviations. We limited the x-axis of step-by-step data to the participant with the fewest number of steps taken during the split-belt phase (done for visual purposes only; all quantitative analyses were conducted on each participant’s full data). Early bins in both groups were significantly less negative (i.e., more symmetrical) in both groups as more trials were completed. We found a significant group by trial interaction for middle bins, indicating that old adults continuously reduced mid-trial asymmetry across all three trials whereas young adults appear to have accomplished this after the first normal trial.

**Fig. 3:**
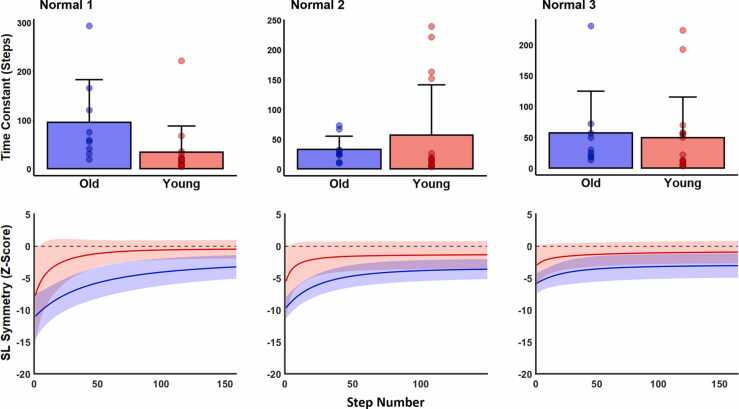
SL_sym_ bins (top row of panels) and step-by-step data traces (bottom row of panels) for Normal 3, BWS, and VISION trials. BWS and VISION represent bodyweight support and visually constricted trials, respectively. In all panels, blue data represents old and red data represents young adults. In step-by-step data, solid lines represent group mean and shaded areas represent standard deviations. We limited the x-axis of step-by-step data to the participant with the fewest number of steps taken during the split-belt phase (done for visual purposes only; all quantitative analyses were conducted on each participant’s full data). The only significant finding from our sensory manipulation analysis was in young adults, whose end-of-trial asymmetry (late bin) remained larger (more negative) in the BWS condition compared to Normal 3.

For this analysis, we were interested in assessing both magnitudes and rates of locomotor adaptation. To define *magnitudes of adaptation*, we used a “binning” method often described in the split-belt treadmill literature. SL_sym_ data from each participant were averaged over the following “bins”: the first 10 strides of split-belt phase (“Split - Early”), the middle 20 strides of the split-belt phase (“Split - Mid”), and the final 20 strides of the split-belt phase (“Split - Late”). This method provides a means to assess the extent to which an individual was affected by the introduction of split-belt walking (i.e., Split - Early) and the general magnitude of adaptation across different bins (i.e., Split – Mid and Split - Late). Bins and mean SL_sym_ data traces for both groups across all trials are provided in Fig. 2, Fig. 3.

As is the case for most split-belt treadmill walking studies, previous age-related research in this field has quantified adaptation using the “binning” method described above. Such comparisons are useful in determining the extent to which individuals or groups adapted over rough periods of time (i.e., the magnitude of adaptation that has occurred), but they do not provide information regarding the precise time course of adaptation. As noted in the introduction, *rate of adaptation* to the split-belt environment provides a behavioral measure of sensorimotor integration and may reveal age-related deficits in locomotor adaptation that would not be observed using the less granular binning method. To more precisely estimate the rate of locomotor adaptation in each trial, we fit a single exponential function to SL_sym_ values during the split-belt phase:

$$y=Ae^{\left(-n/\tau \;\right)}+B$$

Where A and B are free parameters, τ is the time-constant, and n is step number. B was constrained to ± 1 SD of the last 20 steps of the split-belt phase – this constraint allowed the function to decay toward the mean SL_sym_ of the final steps of the split-belt phase rather than toward zero. The time constant, τ, was used to quantify adaptation rate. In this model, larger values of τ represent longer decay time and thus slower adaptation rates.

### 2.4 Statistical analysis

Prior to performing our primary analysis, we first compared each bin (early, mid, late) and adaptation rates across all three normal trials using two-way (trial, group) repeated measures ANOVAs. These were conducted for two reasons. First, they allowed us to determine whether differences in adaptation magnitudes and adaptation rates existed between younger and older adults under “normal” conditions. Second, they allowed us to account for potential trial-to-trial “carry-over” due to repeated exposures in our primary analysis (comparing normal and sensory manipulated trials). To address our primary hypothesis, we used a two-way (condition, group) repeated measures ANOVA to compare adaptation magnitude and adaptation rate between young and old adults across the third normal trial (trial #5) and the sensory manipulated conditions. In these analyses, significant interactions would indicate that a change in condition had a disproportionate effect on one of the groups. The third normal trial was used in these analyses to best account for potential carry-over effects due to repeated exposures to the split-belt environment. Because the early, middle, and late bins are related to one another, Bonferroni adjustments were made to correct for multiple comparisons. In cases of significant interactions from the two-way ANOVAs, post-hoc pairwise comparisons were run with Bonferroni adjustments for multiple comparisons. Because group by condition interactions were used as the benchmark for testing our primary hypothesis, significant main effects were not further analyzed using post-hoc tests. In all statistical analyses, p < 0.05 was used to determine significance and for ANOVAs we provide partial eta squared values ($n_{p}^{2}$) as estimates of effect size. For reference, $n_{p}^{2}$ of 0.01 is considered a small effect size, 0.06 is a medium effect size, and 0.14 or higher is considered a large effect size.

## 3 Results

### 3.1 Protocol completion

Of 17 healthy young adults and 10 healthy old adults enrolled, one young and one old participant were unable to complete the entire protocol and were therefore dropped from all analyses. The young individual found it difficult to maintain position on the treadmill during the visually restricted trial and requested to terminate participation mid-trial. The old adult found split-belt walking uncomfortable and requested to terminate participation during the first exposure to the split-belt environment.

### 3.2 Comparing normal trials

**Fig. 4:**
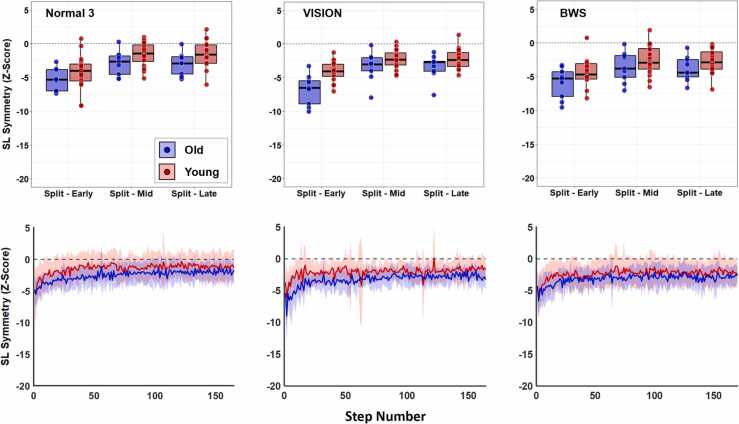
Time constants representing adaptation rate (top row of panels) and exponential fits (bottom row of panels) for all three normal trials. In all panels, blue data represents old and red data represents young adults. For exponential fits, solid lines represent group mean and shaded areas represent standard deviations. We limited the x-axis of exponential fits to the participant with the fewest number of steps taken during the split-belt phase (done for visual purposes only; all quantitative analyses were conducted on each participant’s full data). We found no effects of age, trial number, or their interaction on rates of locomotor adaptation.

The group by trial ANOVA comparing the three normal trials revealed a significant main effect of trial on early bins (df = 2, F = 36.91, p < 0.001, $n_{p}^{2}$ = 0.591) but no group (df = 1, F = 1.55, p = 1.00, $n_{p}^{2}$ = 0.029) or interaction (df = 2, F = 1.44, p = 1.00, $n_{p}^{2}$ = 0.053) effects (Fig. 2). For middle bins, we found no main effect for group (df = 1, F = 4.54, p = 0.396, $n_{p}^{2}$ = 0.087) and a significant interaction (df = 2, F = 6.37, p = 0.036, $n_{p}^{2}$ = 0.211). Whereas younger adults showed no significant difference in middle bins from trial 1 to trial 3 (−0.83 ± 2.30 versus −1.19 ± 1.51 SL_sym_, p = 1.00), older adults significantly reduced asymmetry as more normal trials were completed (−3.56 ± 1.90 versus −1.95 ± 1.27 SL_sym_, p = 0.014). For late bins, we found no significant main effects (group: df = 1, F = 3.22, p = 0.77, $n_{p}^{2}$ = 0.062; trial: df = 2, F= 1.65, p = 1.00, $n_{p}^{2}$ = 0.064) or interaction (df = 2, F = 0.82, p = 1.00, $n_{p}^{2}$ = 0.033). Using the exponential fit analysis, we found no significant main effects (group: df = 1, F = 0.68, p = 0.418, $n_{p}^{2}$ = 0.011; trial: df = 2, F = 0.53, p = 0.594, $n_{p}^{2}$ = 0.017) or interaction (df = 2, F = 2.68, p = 0.079, $n_{p}^{2}$ = 0.081; Fig. 4) for adaptation rates across the three normal trials.

### 3.3 Normal versus sensory manipulated trials

**Fig. 5:**
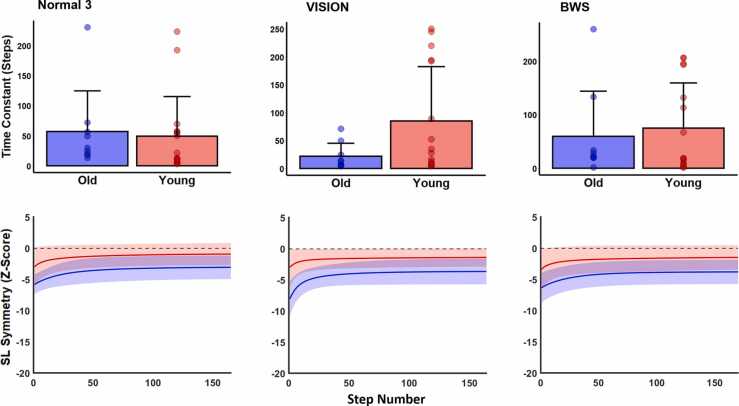
Time constants representing adaptation rate (top row of panels) and exponential fits (bottom row of panels) for Normal 3, BWS, and VISION trials. BWS and VISION represent bodyweight support and visually constricted trials, respectively. In all panels, blue data represents old and red data represents young adults. For exponential fits, solid lines represent group mean and shaded areas represent standard deviations. We limited the x-axis of exponential fits to the participant with the fewest number of steps taken during the split-belt phase (done for visual purposes only; all quantitative analyses were conducted on each participant’s full data). We found no effects of age, sensory condition, or their interaction on rates of locomotor adaptation.

The group by trial ANOVA comparing the third normal trial with the two sensory manipulated conditions revealed no significant main effects (group: df = 1, F = 4.02, p = 0.513, $n_{p}^{2}$ = 0.072; trial: df = 2, F = 1.76, p = 1.00, $n_{p}^{2}$ = 0.063) or interaction (df = 2, F = 2.93, p = 0.567, $n_{p}^{2}$ = 0.101) effects on early bins (Fig. 3). For middle bins, we found no main effect of trial (df = 2, F = 3.60, p = 0.315, $n_{p}^{2}$ = 0.123) or group (df = 1, F = 1.17, p = 1.00, $n_{p}^{2}$ = 0.022) or interaction (df = 2, F = 0.33, p = 1.00, $n_{p}^{2}$ = 0.013) effects. For late bins, we found a significant main effect of trial (df = 2, F = 7.58, p = 0.001, $n_{p}^{2}$ = 0.230), but no group (df = 1, F = 1.71, p = 1.00, $n_{p}^{2}$ = 0.033) or interaction (df = 2, F = 0.48, p = 1.00, $n_{p}^{2}$ = 0.019) effects. Using the exponential fit analysis, we found no significant main effects (group: df = 1, F = 1.48, p = 0.236, $n_{p}^{2}$ = 0.022; trial: df = 2, F = 0.26, p = 0.772, $n_{p}^{2}$ = 0.008) or interaction (df = 2, F = 1.33, p = 0.273, $n_{p}^{2}$ = 0.039; Fig. 5) on adaptation rates across normal and sensory manipulated trials.

## 4 Discussion

The primary purpose of this study was to assess the effects of sensory manipulations on locomotor adaptation to split-belt treadmill walking in both young and old adults. We hypothesized that sensory manipulations would have disproportionately larger effects on locomotor adaption in old compared to young adults, likely due to age-related dysfunction of processes involved in sensorimotor integration. Contrary to this hypothesis, we found no evidence that either proprioceptive or visual manipulations disproportionately influenced adaptation magnitudes or rates in old versus young adults. Our results suggest that both groups were able to appropriately reweight sensory information to maintain locomotor adaptation rates in the face of the sensory manipulations employed here.

As part of our experimental protocol, we had all participants perform three split-belt trials under normal sensory conditions. While this was done primarily to remove the potential effects of trial-to-trial retention from our primary analysis, it also allowed us to test the effect of acutely repeated exposures to the split-belt environment in both young and old adults. We found that the onset of split-belt walking elicited ~60% smaller initial asymmetries (i.e., less negative early bins) in both groups as more normal trials were completed (i.e., from normal #1 to normal #3). The significant group by trial interaction for mid-trial asymmetry (i.e., middle bins) suggests that old compared to young adults required more exposures to the split-belt environment to achieve similar magnitudes of adaptation by mid-trial. However, we found no differences in adaptation rates either between groups within each trial or within groups across trials when measured using the time constant of a single exponential function. Combined, these results suggest that, although older adults can achieve similar rates of adaptation within each trial, they do not “carry-over” learning across trials to the same extent as young adults. Interestingly, the opposite has been observed in a manual reaching task, where older adults exhibited decreased within-trial adaptation but similar across-trial adaptations compared to young adults (Seidler, 2007). Our results are more consistent with previous research showing that old adults learned a sequence skill task at a similar rate as young adults during practice but had lower consolidation (i.e., poorer task performance) during follow-up testing (Brown, Robertson, & Press, 2009). Others have shown an age-related reduction in skill consolidation when learning new functional skills (Tunney et al., 2004). It should also be noted that at least one previous study reported reduced magnitude and rate of adaptation to split-belt treadmill walking in older versus younger adults during a single exposure to the split-belt condition (Bruijn et al., 2012). Our results are somewhat inconsistent with these previous findings, as we observed no significant differences in adaptation rates and only slight differences in adaptation magnitudes (i.e., interaction of group and trial on middle bins) between younger and older adults. However, Bruijn et al. (2012) used a method similar to the binning calculation employed in our study to quantify rates of adaptation, which we argue provides a less precise measure of adaptation rate. Inconsistent findings on age-related changes to skill acquisition and motor learning are likely explained, at least in part, by the diverse effects of aging in the older adult population at large as well as between-study differences in motor learning tasks and the metrics used to quantify outcomes. High variability in cognitive and physical function within the older adult population at large can create between-study differences in sample characteristics, which may lead to inconsistent study results.

Age-related changes to peripheral sensory receptors and central sensorimotor neural circuits led us to hypothesize that sensory manipulations would have disproportionately larger negative consequences for locomotor adaptation in older adults (Chaput and Proteau, 1996, Degardin et al., 2011, Henry and Baudry, 2019, Piitulainen et al., 2018). Contrary to this hypothesis, we found similar adaption magnitudes and rates between younger and older adults across sensory manipulated trials (i.e., no significant interactions were observed). These results are consistent with previous work showing that adaptability is preserved in advanced age during manual reaching tasks with visuomotor manipulations (Bock & Schneider, 2002). There is also consistent and compelling evidence that healthy older adults maintain a high capacity to adapt gait mechanics. For example, healthy aging is associated with a distal-to-proximal (ankle-to-hip) redistribution of joint-level kinetics (Beijersbergen et al., 2017, DeVita and Hortobagyi, 2000, Franz, 2016, Franz and Kram, 2014, Kuhman et al., 2018). Although this phenomenon was traditionally considered maladaptive, emerging evidence suggests that it is beneficial in nature (Beijersbergen et al., 2017, Krupenevich and Miller, 2021, Kuhman et al., 2018, Kuhman et al., 2018). Evidence of motor adaptability has also been observed kinematically during more complicated walking tasks. Specifically, during “cued” walking on a treadmill - where individuals must place their feet on targets specified by lighted cues - older adults exhibit high levels of task-oriented inter-segment covariation from step to step, indicating the use of “motor flexibility” (Rosenblatt, Eckardt, Kuhman, & Hurt, 2020). The convergence of results across a variety of experimental and analytical approaches strengthens the notion that healthy aging does not limit motor adaptability, at least as it relates to locomotion. However, it is important to note that, at least to our knowledge, no study has shown adaptability across different experimental/analytical approaches within the same cohort.

**Fig. 6:**
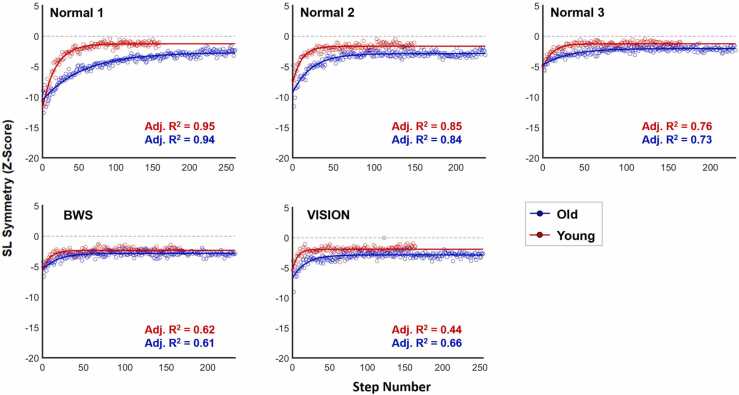
Exponential functions applied to group-averaged data for both young and old adults in each condition. The exponential model fit group-averaged data well, as evidenced by the adjusted R-squared values in each panel. When applied to individual participant data, model fits varied from subject to subject, as evidenced by shaded regions in the bottom row of panels in Fig. 3, Fig. 5. All statistical analyses were conducted using data from model fits to individual participant data.

It is important to consider several limitations when interpreting our findings. First, individuals completed only five minutes of split-belt treadmill walking in each condition. Although we observed the same kinematic adaption typically observed in split-belt protocols, emerging evidence suggests longer-term adaptations occur well beyond five minutes (Sánchez, Simha, Donelan, & Finley, 2021); our results are therefore limited to acute, shorter-term adaptation. Our condition randomization process may have influenced results. Although we randomized order of sensory manipulations, our normal trials were always completed first, second, and last. In hindsight, including Normal trials 2 and 3 in the randomization process may have added rigor to our experimental protocol. We used step length symmetry to explore locomotor adaptation. Although this is a common metric in the split-belt literature, other metrics have also been used (e.g., double support time) and may provide further insights into adaptation under sensory manipulated conditions. Similarly, while we used a single exponential equation to model adaptation and calculate rates of locomotor adaptation, it is important to note that other models have been used for similar purposes (Malone and Bastian, 2010, Mawase et al., 2013, Roemmich et al., 2016). Although our single exponential model fit group-averaged data well (Fig. 6), individual model fits varied from subject to subject. We applied several other published models/methods (and even developed one of our own) to our data and were unable to achieve significantly better fits compared to the relatively simple, single exponential model. Our sample of older adults was relatively small, which limits our statistical power and thus our ability to draw firm conclusions from our results. The older adults included in this study were healthy and high functioning (e.g., no serious health conditions, independently mobile, able to successfully complete this relatively challenging protocol). Our results therefore may not translate to the old adult population at large, which includes lower functioning but clinically non-impaired individuals. Unfortunately, but understandably, issues related to the COVID-19 pandemic made it difficult to recruit and enroll older participants into this study. Our primary hypothesis was driven in large part by the assumption that older adults would have deficits in the neural organs and processes underpinning motor adaptation. Unfortunately, we did not perform proprioception or visual tests and therefore do not know whether previously reported deficits in older adults at large existed in our relatively small cohort. It is possible that the sensory manipulations used in this study were not sufficient to cause observable differences in adaptation. Sensory losses greater than 30% BWS, less than 20° visual field, or via multiple manipulations simultaneously may be required to detect adaptation differences in healthy older adults. It is also possible that alternative sensory manipulations (e.g., tendon vibration) would have altered our results. Our BWS system may have altered adaptation in ways that are not necessarily related to reduced proprioception. For example, both stability and energetic cost are likely important drivers of adaptation. Our BWS system may have provided actual or perceived stability for some individuals (e.g., by reducing fear of falling) or altered the energy landscape of the walking environment in ways that affected the adaptation process.

## 5 Conclusion

In conclusion, we found that externally manipulated proprioception and vison did not disproportionately effect locomotor adaptation magnitude or rate in healthy older compared to younger adults. Our results provide behavioral evidence that both groups were able to adequately reweight sources of sensory information based on environmental constraints, indicative of well-functioning sensorimotor integration.

## CRediT authorship contribution statement

**Daniel Kuhman**: Conceptualization, Data curation, Formal analysis, Investigation, Methodology, Software, Validation, Visualization, Writing – original draft, Writing – review & editing. **Alyson Moll**: Investigation, Methodology, Writing – original draft, Writing – review & editing. **William Reed**: Conceptualization, Methodology, Project administration, Writing – original draft, Writing – review & editing. **Noah Rosenblatt**: Conceptualization, Formal analysis, Methodology, Project administration, Supervision, Writing – original draft, Writing – review & editing. **Kristina Visscher**: Conceptualization, Methodology, Project administration, Writing – original draft, Writing – review & editing. **Harrison Walker**: Conceptualization, Methodology, Project administration, Writing – original draft, Writing – review & editing. **Christopher P. Hurt**: Conceptualization, Data curation, Formal analysis, Investigation, Methodology, Project administration, Resources, Software, Supervision, Validation, Visualization, Writing – original draft, Writing – review & editing.

## Declaration of Competing Interest

The authors have no conflicts of interest to declare relating to this work.
